# Case series of *Bartonella quintana* blood culture-negative endocarditis in Washington, DC

**DOI:** 10.1099/jmmcr.0.005049

**Published:** 2016-08-30

**Authors:** Fisseha Y. Ghidey, Osamuyimen Igbinosa, Kristin Mills, Leon Lai, Christian Woods, Maria E. Ruiz, Dawn Fishbein, Rahul Sampath, Robert Lowery, Glenn Wortmann

**Affiliations:** ^1^​ Infectious Disease Section, MedStar Washington Hospital Center, Washington, USA; ^2^​ Mayo Clinical Laboratory, Rochester, MN, USA

**Keywords:** *Bartonella quintana*, endocarditis, Culture-negative

## Abstract

**Introduction:**

Prior studies (predominantly from Europe) have demonstrated blood culture-negative endocarditis due to *Bartonella*. Our objective was to describe three cases of *Bartonella quintana* endocarditis identified within one year at a large hospital in Washington, DC, USA.

**Case presentation:**

We constructed a descriptive case series from a retrospective review of medical records from April to December 2013 at an 800-bed urban hospital. All three patients (ages: 52, 55 and 57 years) were undomiciled/homeless men with a history of alcoholism. Although they had negative blood cultures, echocardiography demonstrated aortic/mitral valve perforation and regurgitation in one patient, aortic/mitral valve vegetation with mitral regurgitation in the second patient, and aortic valve vegetation with regurgitation in the third patient. The patients had positive *Bartonella quintana* serum immunoglobulin G (IgG) with negative immunoglobulin M (IgM). PCR on DNA extracted from cardiac valves was positive for *Bartonella*, and DNA sequencing of PCR amplicons identified *Bartonella quintana*. Patients received treatment with doxycycline/rifampin or doxycycline/gentamicin.

**Conclusion:**

Clinicians should consider *Bartonella* endocarditis as a differential diagnosis in patients who fit elements of the Duke Criteria, as well as having a history of homelessness and alcoholism.

## Introduction

*Bartonella quintana* is a small, fastidious Gram-negative rod that caused trench fever during World War I. The organism can lead to chronic bacteraemia and endocarditis among the homeless and in patients who chronically abuse alcohol (Foucalt *et al.*, 2002; Fournier *et al.*, 2001). The human body louse (*Pediculus humanus*) is a known vector for* Bartonella* (Ohl & Spach, 2000). *Bartonella* is considered an uncommon aetiology of infective endocarditis as it is reported in only 3 % of cases in the literature (Lamas & Eykyn, 2003). In 1993, two separate reports first implicated *Bartonella* in endocarditis (Rolain *et al.*, 2004; Spach *et al.*, 1993), and over the years, the bacteria became more appreciated as a significant cause of blood culture-negative endocarditis (Daly *et al.*, 1993).

*Haemophilus*, *Aggregatibacter*,* Cardiobacterium*,* Eikenella* and *Kingella* (HACEK) organisms more commonly cause blood culture-negative endocarditis, and they are easily isolated when the bacteria are incubated for at least five days. Hence, ‘true’ blood culture-negative endocarditis is due to other fastidious organisms, notably *Coxiella burnetii* and *Bartonella* spp. Six species of *Bartonella* can cause infective endocarditis in humans: *Bartonella quintana, B. henselae, B. elizabethae, B. vinsonii*,* B. koehlerae *and* B. alsatica* (Rolain *et al.*, 2004; Spach *et al.*, 1993; Daly *et al.*, 1993). Of note, *B. quintana* and *B. henselae* have been associated with over 95 % of culture-negative infective endocarditis. Due to difficulties in culturing *Bartonella* from blood, diagnosis typically requires serological and molecular methodologies. The two most commonly used serological methods for *Bartonella* infections are indirect immunofluorescence assay (IFA) and enzyme-linked immunosorbent assay (ELISA). Titres of 1 : 1600 (via a microimmunofluorescence test) have a positive predictive value of 0.88 (Daly *et al.*, 1993).

PCR-based tests on blood and cardiac valvular tissue have an important role in the diagnosis of *Bartonella* endocarditis, even in the setting of prior antibiotic use. One study reported positive PCR testing for *Bartonella* on cardiac valve tissue in more than 95 % of patients with *Bartonella* endocarditis (Raoult *et al.*, 1996). Some of the most commonly cited molecular targets for diagnosis of *Bartonella* endocarditis by PCR include 16S rRNA gene, internal transcribed spacer (ITS), RNA polymerase beta-subunit-encoding gene (*rpoB*) and citrate synthase gene (*gltA*). The 16S rRNA gene has been the focus for most PCR methods because it is one of the most conserved genes (Weisburg *et al.*, 1991). However, the 16S rRNA genes of species of the genus *Bartonella* share more than 97.8 % similarity (Birtles, 1995), and as a result, differences between them are not sufficient for needed discrimination between species. PCR amplification targeting the 16S–23S rRNA gene ITS region is considered highly sensitive, but amplification specificity depends on primer design, and because of published concerns about nonspecific PCR amplification of species of the genus *Mesorhizobium* associated with ITS (Maggi & Breitschwerdt, 2005), we elected to use the *gltA* method which is now commercially available at the Mayo laboratory. Citrate synthase is the first enzyme of the tricarboxylic acid cycle and thus is a key regulator of intracellular adenosine triphosphate production in both prokaryotic and eukaryotic cells (Wiegand & Remington, 1986) The use of *gltA* comparisons to estimate divergence among closely related species was first proposed by Regnery and his colleagues, who demonstrated genotypic relationships among species of the genus *Rickettsia* by comparing multiple restriction endonuclease digestion patterns of partial *gltA* PCR amplicons (Regnery *et al.*, 1991).

In the past decade, we have dramatically increased our awareness and understanding of human disease caused by *Bartonella* spp. Investigators from France have generated the majority of reports on endocarditis due to *Bartonella*, including several multi-centre international studies with patients from France, England and Canada ; Fournier *et al.*, 2001; Daly *et al.*, 1993). Until now, only one report of *B. quintana* bacteraemia (with and without endocarditis) in homeless persons without human immunodeficiency virus (HIV) infection had been reported in the USA, specifically in Seattle, WA (Spach *et al.*, 1993).

We report three cases of *B. quintana* endocarditis that occurred within one year at our hospital in Washington, DC. We also compare our data to the increasing number of blood culture-negative endocarditis cases reported in the literature.

## Methods

We performed a retrospective review of medical records after receiving Institutional Review Board (IRB) approval. Patients were evaluated by the infectious diseases consultation service, and laboratory testing for *Bartonella* was ordered as part of the clinical evaluation. The diagnosis of *Bartonella* endocarditis was based on the modified Duke criteria (Li *et al.*, 2000) and microbiological findings (including serological and PCR results). *Bartonella *serological and PCR assays were performed at Mayo Medical Laboratories in Rochester, MN. Serology for *Bartonella* was performed with an IFA technique using antigen substrate slides consisting of two separate cell cultures infected with *Bartonella henselae* or *Bartonella quintana *as described by Maurin & Raoult (1996). An immunoglobulin M (IgM) titre of >1 : 20 was suggestive of a current infection with either *B. henselae or B. quintana*, while an immunoglobulin G (IgG) titre of >1 : 128 suggested a current or previous infection. PCR testing was performed on DNA extracted from cardiac valve tissue. A portion of the citrate *gltA* of *B. henselae* served as a target sequence, and a base substitution in the donor probe sequence was used to achieve greater separation in the melting-curve analysis between *B. henselae* and *B. quintana.* (Cockerill & Uhl, 2002). This assay was capable of identifying the genus *Bartonella*, but could not reliably distinguish among the various species. DNA sequencing of PCR amplicons was performed using the Promega gel purification kit and two picomoles of forward and reverse primers. Sequencing was performed with the Big Dye sequencing method on an Applied Biosystems 3130 genetic analyzer, and the resulting sequence was queried via the Basic Local Alignment Search Tool (blast) for species identification (Avidor *et al.*, 2004).

## Results

Three cases of infective endocarditis caused by *B. quintana* were identified between April and December 2013 (Table 1). All three patients were homeless and had a history of alcoholism. Blood cultures were negative on multiple occasions for all patients, but echocardiography demonstrated aortic/mitral valve perforation and severe regurgitation in one patient, aortic/mitral valve vegetation with severe mitral regurgitation in the second patient, and large aortic valve vegetation with severe regurgitation in the third patient. *B. quintana* and *B. henselae* IgM antibody titres were negative for all patients. Patient 1 had *B. henselae* and *B. quintana* IgG titres of >1 : 1024, as well as a *Chlamydophila pneumonia* IgG of 1 : 256. Patient 2 had a *B. henselae* IgG titre of >1 : 1024 and a *B. quintana* IgG titre of 1 : 512. Patient 3 had a *B. henselae* IgG titre of >1 : 1024 and a *B. quintana* IgG titre of <1 : 128.

**Table 1.** Characteristics of three patients with *Bartonella quintana* infective endocarditis AV, Aortic valve; MV, mitral valve; +, positive.

Patient no.	Age (years)/sex	Risk factors	*B. henselae *serology	*B. quintana* serology	Affected valve	Valve PCR of *gltA*
1	52/male	Homeless, alcoholism	IgM: negative IgG: >1 : 1024	IgM: negative IgG: >1 : 1024	AV/MV	+
2	55/male	Homeless, alcoholism	IgM: negative IgG: >1 : 1024	IgM: negative IgG: 1 : 512	AV/MV	+
3	57/male	Homeless, alcoholism	IgM: negative IgG: >1 : 1024	IgM: negative IgG: <1 : 128	AV	+

Due to cardiac valve destruction or incompetence, valve replacement was required for all patients. DNA extracted from cardiac valve tissue was identified by PCR as part of the genus *Bartonella*, and subsequent DNA sequencing and blast alignment revealed 99 % sequence similarity with *B. quintana* for the three cases. All patients received doxycycline for six weeks. Two patients received concomitant rifampin for two weeks, while one patient received gentamicin for one week (but it was changed to rifampin for an additional week due to aminoglycoside-induced nephrotoxicity).

## Discussion

Species of the genus *Bartonella* are fastidious Gram-negative bacteria that require specific laboratory conditions for laboratory growth and identification. In our case series, we observed a cross-reaction between *Bartonella *and *Chlamydophilia *in one patient. Identification of *Bartonella *species with serology was unreliable in our patients, as *B. henselae* IgG titres were higher than *B. quintana* titres in two patients, and equivalent in one patient. All of our patients had detectable *Bartonella* DNA from cardiac valve tissue, and even though PCR of valve tissue reportedly has 100 % specificity, the sensitivity of this test can be compromised with prior antibiotic use (Fournier *et al.*, 2010). DNA sequencing of the amplified PCR products and comparison with blast submissions allowed us to establish *B. quintana* as the causative bacteria in our cohort.

Similar to other cases reported in the literature, our patients were homeless, abused alcohol, and ultimately required cardiac valve replacement. In a report of 101 patients with *Bartonella *endocarditis, Raoult *et al*. (2003) reported that 85 % were male, 38 % were homeless, 48 % were alcoholic, and 75 % underwent valvular surgery because of severe valve damage at the time of diagnosis (Raoult *et al.*, 2003). Due to the limited number of cases, the optimum treatment for suspected or confirmed* Bartonella* endocarditis is unclear, but current recommendations suggest doxycycline for six weeks plus gentamicin sulfate for two weeks. In our cohort, all three patients eventually received treatment with doxycycline/rifampin.

In summary, although much of the existing literature regarding *Bartonella *endocarditis stems from Europe, our findings suggest that *Bartonella *endocarditis should also be considered in the USA (particularly in patients who are homeless and have a history of alcoholism). Papers by both Fournier *et al and Beitkopt et al * have developed an algorithm that suggests a diagnostic strategy for evaluating blood culture-negative endocarditis and recommends serological testing for *Bartonella* (Fournier *et al.*, 2010; Breitkopf *et al.*, 2005). Based on our case series, we concur with that recommendation, and suspect that *Bartonella* endocarditis may be an under-recognized cause of blood culture-negative endocarditis in the USA. Our improved knowledge of the spectrum of pathogens involved in blood culture-negative endocarditis should also advance our empiric antibiotic therapy selections.
